# Periodontal diseases in Thai schoolchildren. Clinical and microbiological observations

**DOI:** 10.1007/s10266-023-00817-w

**Published:** 2023-05-08

**Authors:** Supacharin Piwat, Amina Basic, Nuntiya Pahumunto, Rawee Teanpaisan, Gunnar Dahlen

**Affiliations:** 1 Common Oral Diseases and Epidemiological Research Center, Faculty of Dentistry, Prince of Songkla University, Hat Yai, Thailand; 2 Department of Preventive Dentistry, Faculty of Dentistry, Prince of Songkla University, Hat Yai, Thailand; 3 Department of Oral Microbiology and Immunology, Institute of Ododntology, Sahlgrenska Academy, University of Gothenburg, Box 450, 40530 Gothenburg, Sweden

**Keywords:** Periodontitis, Early onset periodontitis, Adolescents, Thai schoolchildren, Subgingival microbiota

## Abstract

The prevalence of periodontitis among Thai schoolchildren is unknown. In a cross-sectional study, the prevalence and severity of periodontal diseases, in a group of Thai schoolchildren, along with the presence and numbers of bacterial species commonly associated with periodontitis were investigated. A consent form was sent out to 192 schoolchildren in one school (Chanachanupathom School) in Chana, Southern Thailand (in the age range of 12–18 years) and 119 attended for a clinical and microbiological examination. Clinical recordings included number of teeth present, DMFT, plaque index, bleeding index, clinical attachment loss (CAL), and probing pocket depth (PPD). Pooled plaque samples were analyzed with culture and qPCR against bacteria associated with periodontitis. The children had low caries experience (DMFT = 3.2 ± 2.3), poor oral hygiene, high bleeding scores, and 67 (56.3%) had at least one interproximal site with CAL ≥ 1 mm. Thirty-seven (31.1%) of the children were diagnosed with periodontitis stage I, and sixteen (13.4%) were classified as periodontitis Stage II. *Aggregatibacter actinomycetemcomitans* was sparsely found in all but the healthy clinical groups (gingivitis, periodontitis Stage I and II), while the groups showed a high prevalence of *Fusobacterium* spp.*, Prevotella intermedia/nigrescens*, and *Campylobacter* species as well as of the periodontitis-associated species *Porphyromonas gingivalis, Treponema denticola*, and *Tannerella forsythia.* Thai schoolchildren have poor oral hygiene with abundant amounts of plaque and high presence of bleeding. Early onset periodontitis is common but mostly in its mild form and is not associated with the presence of *A. actinomycetemcomitans*.

## Introduction

Periodontal diseases are chronic inflammatory diseases induced by microorganisms of the dental plaque. Gingivitis is characterized by inflammation in the gingival tissues, while also a progressive destruction of the tooth supporting tissues is seen in periodontitis. The prevalence of periodontitis is increasing with increasing age, due to the fact that the destructions are cumulative. The incidence rate has been reported to be highest at 30–40 years of age [1]. Around 10% of the populations worldwide are reported to suffer from periodontitis in its severe forms, leading to advanced periodontal breakdown and loss of teeth after the age of 50 [2]. However, the age of onset of periodontitis is still unclear, although Thorbert-Mros proposed 22–28 years to be the onset for severe periodontitis [3]. Principally, periodontitis may start at any age and also to involve teeth of the primary dentition [4].

The classification of periodontitis has recently been revised [5, 6]. According to the new consensus, periodontitis should be classified in Stages (I–IV), defined by severity, complexity, extent, and distribution. The severity should be based on the level of interdental clinical attachment loss, presence of furcation involvements, and angular bony defects among other parameters. The term “aggressive periodontitis”, previously used as a separate clinical diagnosis for early and rapid breakdown primarily in young individuals is no longer used [6, 7].

Few studies have addressed the initial stages (early onset of the disease) because of the uncertainty in CAL measurements at the early stage. Commonly ≥ 3 mm CAL, preferentially on several sites and together with visible radiographic bone loss (if radiographs are available) has been used for case definition [3, 8]. It means that the disease starts years earlier and Thorbert-Mros et al. [3] reported in adults having severe generalized periodontitis at 35–45 years of age that the disease commenced mainly between 22 and 28 years of age. On the other hand, Lopez et al. [8] using CAL measurements of ≥ 1 mm indicated that periodontal breakdown is commonly present already among adolescents.

The prevalence of periodontitis in children and adolescents is generally low but differ significantly between populations. In a systematic review, which included adolescents, Susin et al. reported [9] that in most studies, “aggressive” forms occur in less than 0.5% of the world population (mostly Caucasian populations) with a slightly higher prevalence rates for African countries. The periodontal conditions in the South-East Asian populations are sparsely known. Study from Indonesia by Timmerman et al. [10] reported a prevalence of 26% for CAL ≥ 3 mm in an age group of 15 years old up to 25 years of age; however, the prevalence in the lower age span (15–18 years) was not separately reported.

The prevalence and severity of periodontitis and its association to certain microorganisms could differ significantly between populations. The focus has mainly been on the rapidly progressive forms of periodontitis with a molar/incisor pattern, previously termed localized periodontitis or aggressive periodontitis and *A. actinomycetemcomitans* [11–13], while the prevalence of other periodontitis-associated microorganisms is less well studied in children [14].

Due to the unknown periodontal conditions among children/adolescents of South-East Asia and prevalence of periodontitis-associated bacteria, we decided to perform a cross-sectional observational study to describe the periodontal conditions and the prevalence and severity of periodontal diseases in a group of Thai schoolchildren living in Chana, a suburban area of the city of Hat Yai in Southern Thailand. Since previous investigations in the same population have disclosed a high prevalence of *A. actinomycetemcomitans* among adult periodontitis cases, we hypothesized that this bacterial species would also be associated with early onset periodontitis in children/adolescents [15, 16]. We also aimed to investigate the presence and numbers of other bacterial species commonly associated with periodontitis, using both culture and qPCR for detection.

## Materials and methods

### Study population

The study was conducted together with Chanachanupathom School in Chana, Songkla province in southern Thailand. Teachers were requested to ask all 192 schoolchildren from the classes with children of age 12–18 years to participate in an investigation on the prevalence of periodontitis. Before commencing, the study was approved by The Ethics Committee for Human Research, Faculty of Dentistry, Prince of Songkla University, Thailand. The children participated on voluntary basis and an informed consent from their parents was received. 119 children attended the examination, while 73 children, mostly older boys declined to come and were considered drop-out.

### Clinical registrations

The registrations were performed in the classroom under field conditions using a portable dental chair and spotlight. The clinical registrations were performed by three dentists (author SP and GD, and an experienced dentists from the local department of pediatric dentistry (see Acknowledgements)). The three examiners were carefully instructed prior to the study on how to perform the measurements. Decayed, Missed, Filled Teeth (DMFT) was recorded. Plaque index was registered as % of 12 buccal/lingual sites of 6 teeth (one tooth in each sextant) with visible plaque. Full mouth (permanent teeth) recordings of the patient’s clinical attachment loss (CAL), probing pocket depth (PPD), and bleeding on probing (BoP) were assessed. Special attention was made for the first molars and the incisors, because these teeth have been present in mouth the longest time and most commonly show periodontal breakdown in the localized form of periodontitis [17]. All three parameters (CAL, PPD, BoP) were recorded at four sites per tooth (permanent teeth only); the mesio-buccal, mid-buccal, disto-buccal, and mid lingual aspect of all fully erupted teeth. Probing pocket depth was measured to the nearest whole mm as the distance between the gingival margin and the bottom of the pocket by probing, and the CAL was measured as the distance from the enamel–cement junction to the bottom of the pocket.

### Criteria used for case definition and classification

Teeth with BoP and attachment loss were diagnosed with periodontitis. Interdental attachment loss at two non-adjacent teeth due to periodontitis was set as minimum for the patient to be a periodontitis case. The classification of periodontitis cases was based on the criteria of staging and grading, according to the recent consensus report of the 2017 World Workshop [6]. The stage determinants of periodontitis were based on interdental clinical attachment loss and probing pocket depth (PPD). The patients were classified as Stage I, initial periodontitis, if CAL loss was 1–2 mm and PPD ≤ 4 mm. Stage II were patients with CAL loss of 3–4 mm, or patients with CAL loss of 1–2 mm, and PPD ≤ 5 mm. Patients with no evident CAL loss at two non-adjacent teeth were diagnosed with gingivitis if BoP was ≥ 10% [18].

### Microbial sampling

Pooled microbiological samples were taken from the mesial interproximal site of four teeth (upper and lower central incisors and first molars) with a curette and transformed to a bottle with transport medium VMGAIII [19] for analysis by culture and qPCR. All samples were kept in a low temperature refrigerator during days of examination until transportation. The transportation time was < 24 h. A volume of 100 µl of the VMGAIII medium was immediately subjected for culture at arrival to the Laboratory of Oral Microbiology at the Institute of Odontology, University of Gothenburg, Sweden. The remaining VMGAIII medium was stored in a low temperature freezer until qPCR analysis.

### Microbiological cultivation and identification

The samples were thoroughly mixed and spread evenly onto Brucella (BBL, Cockeysville, MD, USA) blood agar plates supplemented with 1.5% hemolyzed human erythrocytes, and 5 × 10^5^ mg/l menadione. The Brucella agar plates were cultured anaerobically for 7 days in 37 ℃, and the total number of colonies was counted as total viable counts (TVC). The specific bacteria were identified and counted as follows: *Fusobacterium nucleatum* was identified as grayish nacracious colonies and appearing as filamentous Gram-negative rods at Gram staining. Black-pigmented colonies stained as Gram-negative rods were identified based on their fluorescens in UV light either as *Porphyromonas gingivalis* (non-fluorescent) or *Prevotella intermedia/Prevotella nigrescens* (brick red fluorescence) [20]. Gram-negative rods with a colony morphology with a spread and corroding appearance and showing motility was classified as *Campylobacter* spp., while those with a gliding appearance were classified as *Capnocytophaga* species.

In addition, one TSBV agar plate (trypticase-soy agar with bacitracin 75 mg/L and vancomycin 5 mg/L) according to Slots [21] for selective culture of *Aggregatibacter actinomycetemcomitans*, was inoculated. The plates were incubated for 5 days in 37 ℃ and 10% CO_2_. The plates were inspected for colonies with typical *A. actinomycetemcomitans* morphology showing star-shaped or small smooth, adherent and catalase-positive colonies, and with a micro-morphology being Gram-negative coccobacilli. When *A. actinomycetemcomitans* colonies were identified on the agar plate, the isolates were pure cultured and kept at – 80 ℃ until further identification. The presence of the leukotoxin gene (*ltx*) was used to verify species identity of the *A. actinomycetemcomitans* isolates. The PCR product of each strain was run by electrophoresis to identify leukotoxin gene variants including the JP2 genotype [22].

### Microbial quantification with qPCR

The samples were also analyzed with real-time PCR (qPCR) using 16S specific primers according to Kuboniwa et al. [23] against *P. gingivalis*, *Tannerella forsythia*, *Treponema denticola*, *Prevotella intermedia*, *Fusobacterium nucleatum*, *Campylobacter rectus*, *Campylobacter gracilis*, and *A. actinomycetemcomitans.* Briefly, the samples were washed with Tris-buffer and the tubes were boiled for 10 min. The PCR assay was performed in 10 μl of Sso Fast™ EvaGreen® Supermix (Bio-Rad), and 5 μl (1 μM) of each primer and 5 μl of sample were added. The following primers (Invitrogen, Lidingö, Sweden) were used (forward and reverse, respectively): *A. actinomycetemcomitans* 5´-CTA GGT ATT GCG AAA CAA TTT G-3´and 5´-CCT GAA ATT TAA GCT GGT AAT C-3´; *Campylobacter rectus* 5-TTT CGG AGC GTA AAC TCC TTT TC-3´and 5´-TTT CTG CAA GCA GAC ACT CTT-3´; *Campylobacter gracilis* 5´-AAC GGA ATT TAA GAG AGC TT-3`and 5`-CTT TCC CGA TTT ATC TTA TG-3`; *Fusobacterium* spp., 5´-GGA TTT ATT GGG CGT AAA GC-3´ and 5´-GGC ATT CCT ACA AAT ATC TACGAA-3; for *P. gingivalis,* 5´-TGT AGA TGA CTG ATG GTG AAA ACC-3´ and 5´-ACG TCA TCC CCA CCT TCC TC-3´; for *P. intermedia*, 5´-TTT GTT GGG GAG TAA AGC GGG-3´ and 5´-TCA ACA TCT CTG TAT CCT GCG T-3´; for *T. forsythia*, 5´- -3´ and 5´-TGC TTC AGT GTC AGT TAT ACC T-3´; and for *T. denticola*, 5´-TAA TAC CGA ATG TGC TCA TTT ACA T-3´ and 5´-TCAAAGAAGCATTCCCTCTTCTTCTTA-3´.

The cycling protocol of Kubinowa and colleagues was followed [23].

### Statistical analyses

Statistical analyses were performed in IBM SPSS Statistics Software (Version 27, Chicago, IL, USA) or in GraphPad Prism 9.0. The differences between two groups were explored with independent sample *t* test or Chi-square test for independence (with Yates Continuity Correction) and between four groups with one-way between groups ANOVA or Chi-square test for independence. A *p *value of less than 0.05 was considered statistically significant.

## Results

### Description of periodontal conditions

A total of 119 subjects of the 192 enrolled showed up for the examination. The majority of the drop-out subjects were boys (*n* = 58) from the older age groups (15–18 years), resulting in a skewed population with regard to gender with 69% females and 31% males (Table 1) and the age distribution became significantly different (*p* < 0.05) with a mean age of 13.5 years for boys and 14.2 years for girls. The participants had, in general, poor oral hygiene and high bleeding scores. The boys showed little higher DMFT, and plaque scores, but fewer percentages of CAL ≥ 1 mm compared to the girls, but the differences were small and not statistically significant. 44.5% of the children examined were diagnosed with periodontitis. No severe periodontal breakdown was, however, found (only two subjects had an attachment loss of 2 mm). Sites with ≥ 1 mm CAL were not typically distributed, as in the cases of localized forms of periodontitis, but found involving also other teeth apart from incisors and first molars. The clinical parameters in relation to age of the participants showed that the pubertal group (age 12–14, *N* = 86) had significantly higher plaque index (80.7% vs 70.2%, *p* < 0.05)) compared to the post-pubertal group (age 15–18, *N* = 33). However, the older group consisted of significantly more girls than boys (88% vs 62%, *p* < 0.05).

**Table 1:** Clinical parameters of the 119 Thai schoolchildren examined

Variable	Total *N* = 119	Girls *N* = 82	Boys *N* = 37	
Age (mean ± SD)	14.0 ± 1.5	14.2 ± 1.6	13.5 ± 1.0	*
Missing teeth (mean ± SD)	0.5 ± 1.1	0.5 ± 1.2	0.6 ± 1.0	ns
DMFT (mean ± SD)	3.2 ± 2.3	3.1 ± 2.3	3.3 ± 2.3	ns
BoP (mean ± SD)	45.2 ± 20.8	45.4 ± 21.8	44.8 ± 18.6	ns
Plaque index (mean of 6 sites ± SD)	77.8 ± 21.5	74.9 ± 21.8	84.2 ± 19.5	*
Number of children with CAL ≥ 1 mm (%)^a^	67 (56.3%)	49 (59.8%)	18 (48.6%)	ns
Number of children with PPD ≥ 5 mm (%)^b^	16 (13.4%)	11 (13.4%)	5 (13.5%)	ns
Number of children diagnosed with Periodontitis Stage I or Stage II (%)	53 (44.5%)	37 (45.1%)	16 (43.2%)	ns

*p < 0.05. The differences between two groups were explored with independent sample *t* test or Chi-square test for independence (with Yates Continuity Correction)

^a^At least one site with CAL ≥ 1 mm

^b^At least one pocket with PPD (probing pocket depth) ≥ 5 mm

### Disease prevalence and severity

Sixteen children (14.3%) were diagnosed with periodontitis Stage II (Table 2) and an additional 37 (31.1%) were classified with periodontitis Stage I. The periodontitis cases had significantly higher bleeding scores (51.9% and 70.4% BoP, respectively) compared to non-periodontitis group (8.7% and 36.7% BoP for healthy and gingivitis). None of the children had a PPD ≥ 7 mm while 16 (13.4%) had PPD ≥ 5 mm and 67 (56.3%) had CAL ≥ 1 mm (Table 1). Most children were, thus, considered to have gingivitis or mild periodontitis. Only three subjects were classified as healthy based on having BoP at less than 10% of the sites. There was a quite equal distribution of periodontitis cases among the participating boys (43.2%) and girls (45.1%).

**Table 2:** Clinical parameters of the Thai schoolchildren divided by periodontal status

Variable	Healthy *N* = 3	Gingivitis *N* = 63	Periodontitis Stage I *N* = 37	Periodontitis Stage II *N* = 16	
Age (mean ± SD)	14.3 ± 0.6	14.0 ± 1.7	13.7 ± 1.3	14.6 ± 1.2	ns
Missing teeth (mean ± SD)	0.3 ± 0.6	0.6 ± 1.3	0.4 ± 0.9	0.5 ± 0.8	ns
DMFT (mean ± SD)	3.3 ± 0.6	3.1 ± 2.4	3.6 ± 2.4	2.7 ± 2.1	ns
Plaque (mean ± SD)	49.7 ± 14.4	74.6 ± 22.6	81.1 ± 20.0	88.1 ± 14.4	*
BoP (mean ± SD)	8.7 ± 10	36.7 ± 15.2	51.9 ± 17.3	70.4 ± 18.9	***
Mean number of sites with CAL ≥ 1 mm (%)	–	0.4 ± 0.8	10.1 ± 7.5	24.2 ± 13.0	
Mean number of sites with PPD ≥ 5 mm (%)	–	–	–	3.3 ± 2.3	

The differences between the four groups were explored with one-way between groups ANOVA

*p < 0.05

***p < 0.001

### Microbial observations

A total of 111 cases were analyzed microbiologically for the presence and level of common periodontitis-associated bacterial species with culture and qPCR.

**Fig. 1:**
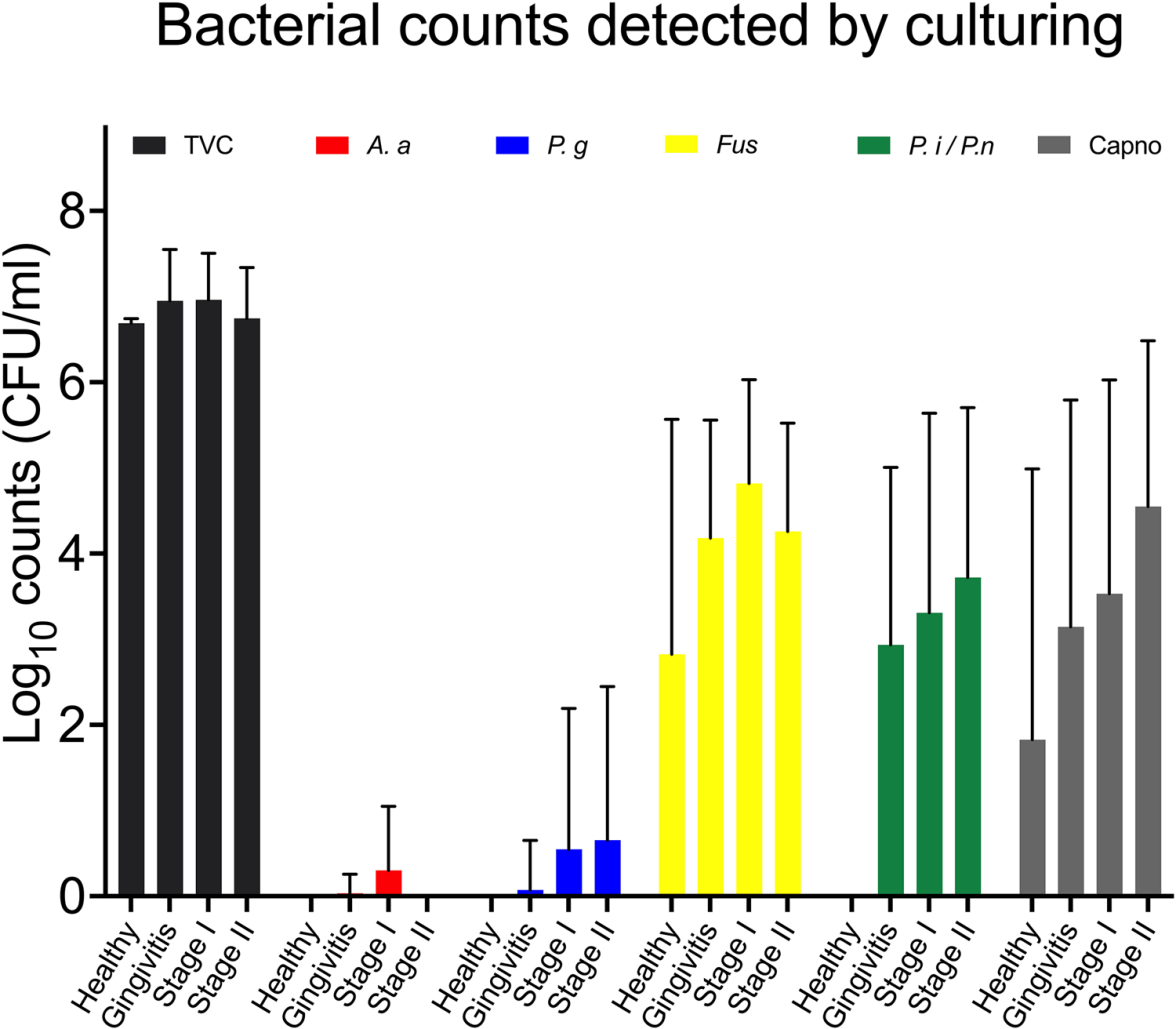
The presence of bacteria associated with periodontitis. The mean log_10_ total viable counts and the mean log_10_ counts of bacterial species associated with periodontal disease are shown

A high frequency in the whole study group was found for *F. nucleatum*, *P. intermedia/P. nigrescens* and for *Capnocytophaga* spp. while *P. gingivalis* was only sporadically detected by culture (Fig. 1). *A. actinomycetemcomitans* was detected on the selective medium (TSBV) in low frequency (7.3%), but 28 samples showed overgrowth of Gram-negative enterics and those plates could not be read.

Although there was a general tendency (Fig. 1) that periodontitis cases (Stage I or II) had higher prevalence and number (counts) of several periodontitis-associated bacterial species as detected by culture, the majority of them did not reach a statistical significance (*p* > 0.05) between the groups. A statistically significant difference was recorded for *P. intermedia/P. nigrescens* when comparing healthy with periodontitis Stage II.

**Fig. 2:**
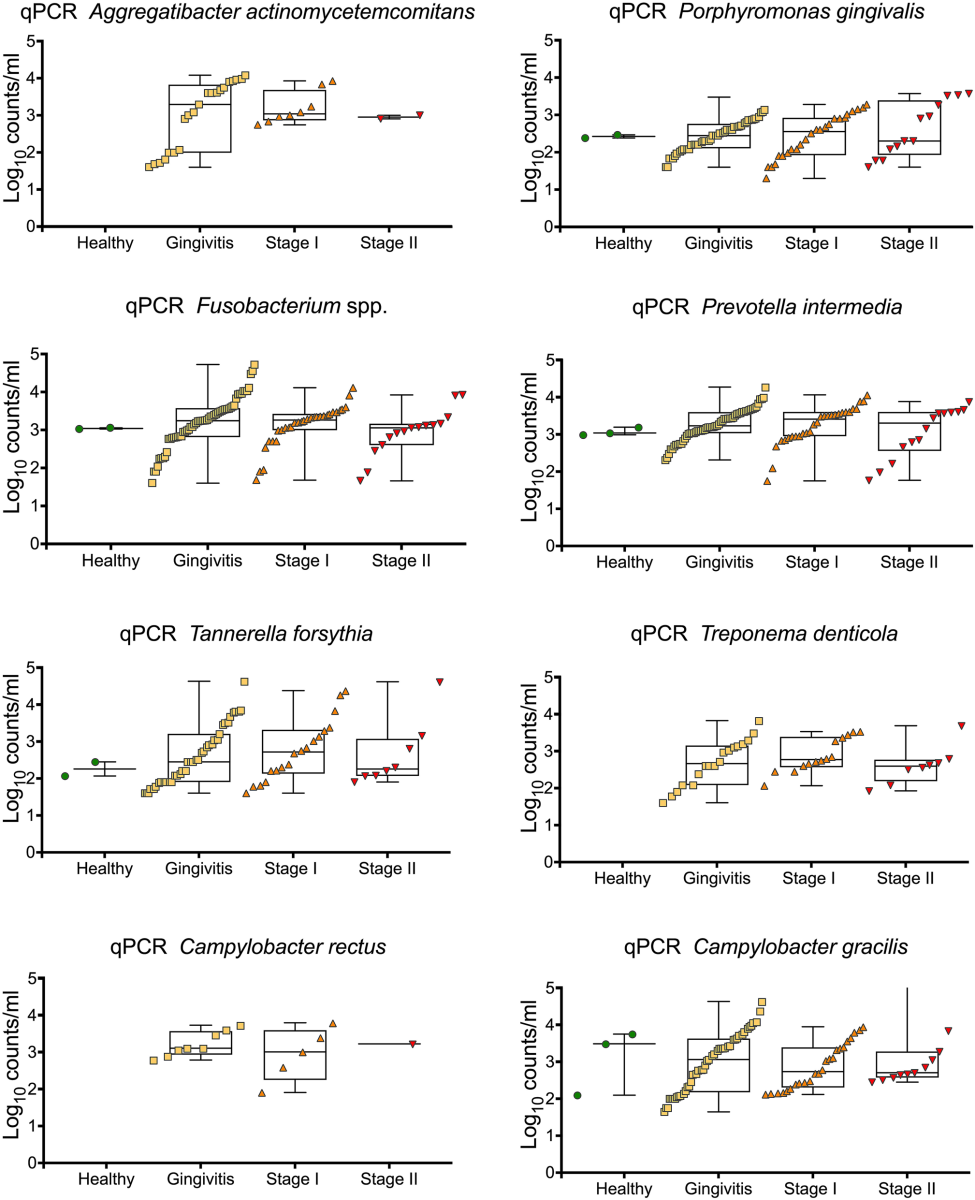
qPCR levels of bacterial species associated with periodontitis. The log_10_ qPCR levels of bacterial species where bacteria were detected are presented, divided in groups based on periodontal status. No statistically significant differences in bacterial number between the four groups were seen

Microbial observations by qPCR, as shown in Table 3 and Fig. 2, show the frequency and mean counts of 8 detected bacterial species associated with periodontitis. A high detection frequency (> 66%) was found for *F. nucleatum, P. gingivalis, C. gracilis*, and *P. intermedia* in all four groups. A high detection rate (50–100%) was also found for *T. forsythia,* while *C. rectus* was low (< 20%) in all groups. *A. actinomycetemcomitans* was detected in none of the healthy cases, in 33.9% of the gingivitis cases, 26.7% of the periodontitis Stage I patients, and 12.5% of the periodontitis Stage II cases. The JP2 clone was not found in any of the samples. The differences between the groups did not reach statistical significance for any of the bacteria detected using qPCR.

**Table 3:** Frequency of subjects positive for bacteria detected with qPCR

Bacteria	Healthy *N* = 3	Gingivitis *N* = 62	Periodontitis Stage I *N* = 30	Periodontitis Stage II *N* = 16	
*A. actinomycetemcomitans* (%)	0 (0)	21 (33.9)	8 (26.7)	2 (12.5)	ns#
*P. gingivalis* (%)	2 (66.7)	44 (71.0)	24 (80.0)	13 (81.3)	ns
*Fusobacterium* spp. (%)	2 (66.7)	57 (91.9)	30 (100)	15 (93.8)	ns
*P. intermedia* (%)	3 (100)	57 (91.9)	28 (93.3)	14 (87.5)	ns
*T. forsythia* (%)	2 (66.7)	39 (62.9)	18 (60.0)	8 (50)	ns
*T. denticola* (%)	0 (0)	18 (29.0)	14 (46.7)	8 (50)	ns
*C. rectus* (%)	0 (0)	8 (12.9)	5 (16.7)	1 (6.3)	ns
*C. gracilis* (%)	3 (100)	45 (72.6)	24 (80.0)	11 (68.8)	ns

ns# = not significant *p* < 0.05. The presence of each bacteria (yes/no) was explored between the four groups with Chi-square test for independence

## Discussion

The present study was conducted to describe the periodontal conditions among Thai schoolchildren at the age of 12–18 of the 119 children that attended the examination. We report a prevalence of 44.5% for initial periodontitis or Stage I periodontitis (31.1%) and Stage II (13.4%) according to the new classification, [6]), where Stage I is defined as having attachment loss of 1–2 mm at two non-adjacent teeth and Stage II also having PPD of 5 mm. None of the subjects examined had CAL loss of  ≥ 3 mm. Poor oral hygiene with abundant amounts of plaque was generally registered and indicated that the oral hygiene was generally not practiced. Gingival inflammation was also generally registered and probing pocket depth (PPD) of 5 mm was found in 13% of the children. Only three individuals were recorded as healthy (BoP < 10%).

The determination of the onset of periodontitis has been a matter of intense debate [8, 24, 25] still not reaching clear consensus. The problem is to find a cutoff level clinically that indicates a true loss of attachment. Clinical and epidemiological studies often use CAL of ≥ 3 mm to be recorded or ≥ 2 mm bone loss to be registered on radiographs (CEJ-BC) before periodontitis can be diagnosed [3, 8, 26–29]. However, the onset of the disease occurs before that and the new consensus considers this by introducing the staging and grading of periodontitis [5, 6]. In epidemiological studies like the present one, radiographs are not always available. The recommendations for case definitions and classification were followed and if a subject had CAL ≥ 1 mm accompanied with BoP on at least two non-adjacent teeth, it was clinically defined as a periodontitis case. Periodontitis cases that had a PPD of 5 mm were classified as Stage II. We like to conclude that the prevalence of early onset periodontitis in this group of Thai schoolchildren was 16 (13.4%) for Stage II. This is within the range of what has previously been found in other South-East Asian populations, e.g., 26% in 15–25 years olds in Indonesia [10] but substantially higher than Caucasians [26, 30, 31] which have a prevalence of less than 5%, but lower than reported in South-American [8, 32] and West-African countries [12, 33]. Except for a few [10, 12, 33], the majority of the above cited studies used radiographical analysis to define periodontal breakdown (CEJ-BC ≥ 2 mm). It is suggested that a lower rate of periodontitis is recorded on radiographs than with only clinical recordings and clinical recordings will overestimate the number of juvenile periodontitis cases in epidemiological studies in which radiographical recordings are not available. We were able to take radiographs using a portable radiographic apparatus (Portable Dental X-Ray System, BEMEMS, Seoul, South Korea) on molars in six clinically severe cases and found bone loss ≥ 2 mm (CEJ-BC) in all of them (data not shown).

Although most studies on the early onset periodontitis deals with adolescents after the puberty and age of 12 [8, 10, 11, 26, 30–32, 34], others have included children of 9 years or even younger [4, 35–37]. However, it must be considered difficult to register CAL and/or radiographical bone resorption in the period were non-erupted or semi-erupted teeth in the dentition are frequent. Interestingly, Sjödin and Matsson [38] reported that 31% of adopted children with Vietnamese origin showed radiographical bone loss when they were 13–19 years old, confirming that periodontal breakdown can start at any age.

Since clinical manifestations of periodontitis are cumulative, the loss of attachment illustrates breakdown experience rather than ongoing disease. This may also be the explanation why the girls in the present study showed more CAL ≥ 1 mm than the boys since the age distribution between became skewed as a consequence that more of the older boys declined to attend. By dividing the subjects in age groups, however, this theory of older adolescents being more diseased does not hold true (number of children with CAL ≥ 1 59.3% for pubertal and 48.5% for post-pubertal age group, respectively, but the difference was not statistically significant). The impact of gender in young populations is low and is probably related to the fact that oral hygiene is less poor in girls compared to boys of the same age [39]. It must also be emphasized that girls in the age of 12–14 years old (around puberty) are biologically more mature (hormonal changes) than boys in corresponding age [40] and may have an onset of periodontitis earlier than boys. Which factor, gender or age, that may be of importance cannot be ruled out in the present study.

The fact that we used both culture and qPCR for specific bacterial detection needs some comments. The possibility of growth of bacteria not sufficiently hampered during transportation is a risk [19]. A moderate overgrowth of Gram-negative enterics on the TSBV agar plates is probably a result of this and the prevalence of *A. actinomycetemcomitans* might be underscored. However, it did not interfere with the readings of Gram-negative anaerobes, which to a large extent survived the transportation and were identified although the proportions could have been altered but still showed a high prevalence of Gram-negative anaerobes confirming the results of the qPCR. By culturing, we were also able to present a total viable count and to save strains for future detailed studies,

*A. actinomycetemcomitans* was expected to be more frequent among the Thai schoolchildren than was found due to the association with periodontitis in adolescents in other populations and the fact that this bacterial species is found almost in all Thai adults [15, 16, 22]. The frequency found in this study with culture (7.3%) or with qPCR (27.9%) is, however, in line with other studies in South-East Asian populations [10, 11, 41, 42]. The Jp2 clone was not detected in any of the children. This was an expected finding since this particular genotype is mainly found in young individuals from Africa and only rarely in Asian populations but hitherto not in the Thai population. This study does not support that A.* actinomycetemcomitans* plays a role as periodontal pathogen in Thai school children.

Gram-negative anaerobes *P. intermedia, F. nucleatum*, *Campylobacter* species and the red complex bacteria *P. gingivalis, T. forsythia, and T. denticola* were frequently detected among the children and with almost no significant differences between the groups. It clearly indicates that the colonization and growth of bacterial species, putatively regarded as periodontal pathogens, already may be present in the subgingival microbiota of Thai schoolchildren where a microbiological dysbiosis is a normality and generally established in young subjects with poor oral hygiene and gingival inflammation. In view of the poor oral hygiene, this observation confirms the findings made in a recent study among Somali child immigrants in Sweden, who showed a high frequency and number of periodontitis-associated bacterial species, implying a more mature and adult type of subgingival microbiota than that of the non-Somali, mostly Swedish children [29].

The clinical and microbiological observations of the periodontal conditions among cross-sectionally recruited children and adolescents are rarely considered from populations with low socioeconomic status, poor attitude to oral hygiene measures, and no organized dental care. The study confirms that early signs of periodontal breakdown and a high frequency and abundance of periodontitis-associated bacteria among children are common and this would predictively lead to severe periodontitis and tooth loss in adulthood. In previous studies conducted in the same province, it was found that severe periodontal disease was present to an extent similar to other populations, although the high prevalence and abundancy of “putative periodontal pathogens” were present in adults 30–39 years of age [43]. All had bleeding (99.9 mean%) of the 30–39 year group, 23.9% had CAL of + 1 mm, but only 2.0% had CAL + 7 mm. In the same population, the microbiology was evaluated with both culture and DNA–DNA hybridization technique (Checkerboard) [15, 16] and showed an almost 100% prevalence and high abundancy of 27 oral bacterial species including *A. actinomycetemcomitans, P. gingivalis, T. denticola*, and other periodontitis-associated bacterial species. We, therefore, want to emphasize that the microbiology is strongly associated with poor oral hygiene and gingival inflammation and this does not necessarily lead to severe periodontal disease in adulthood. Important factors contributing to the transition from gingivitis into progressive periodontitis are still largely unknown. It is believed that the functionality (and not the presence) of the microbiota, in combination with the host response, is of importance for disease progression.

In conclusion, Thai schoolchildren have poor oral hygiene with abundant amounts of plaque and with an almost consistent presence of bleeding on probing and strong association with anaerobic Gram-negative periodontopathogens. Early onset periodontitis is common but in its mild form and not associated with the presence of *A. actinomycetemcomitans*.

## Data Availability

All data supporting the findings of this study are available within the paper.
